# Neuroprotective Transcription Factors in Animal Models of Parkinson Disease

**DOI:** 10.1155/2016/6097107

**Published:** 2015-12-31

**Authors:** François-Xavier Blaudin de Thé, Hocine Rekaik, Alain Prochiantz, Julia Fuchs, Rajiv L. Joshi

**Affiliations:** Center for Interdisciplinary Research in Biology (CIRB), Labex Memolife, CNRS UMR 7241, INSERM U1050, Collège de France, 11 place Marcelin Berthelot, 75231 Paris Cedex 05, France

## Abstract

A number of transcription factors, including En1/2, Foxa1/2, Lmx1a/b, Nurr1, Otx2, and Pitx3, with key roles in midbrain dopaminergic (mDA) neuron development, also regulate adult mDA neuron survival and physiology. Mouse models with targeted disruption of some of these genes display several features reminiscent of Parkinson disease (PD), in particular the selective and progressive loss of mDA neurons in the substantia nigra pars compacta (SNpc). The characterization of these animal models has provided valuable insights into various mechanisms of PD pathogenesis. Therefore, the dissection of the mechanisms and survival signalling pathways engaged by these transcription factors to protect mDA neuron from degeneration can suggest novel therapeutic strategies. The work on En1/2-mediated neuroprotection also highlights the potential of protein transduction technology for neuroprotective approaches in PD.

## 1. Introduction

Parkinson disease (PD) is the second most common neurodegenerative disorder. The disease is characterized by a loss of midbrain dopaminergic (mDA) neurons in the substantia nigra pars compacta (SNpc) and the presence of *α*-synuclein-containing protein aggregates and termed Lewy bodies (and/or Lewy neurites) in affected neurons [1, 2]. Apart from certain familial monogenic forms of the disease, in which mutated genes (e.g.,* SNCA*,* LRRK22*,* PINK1*,* PARKIN*,* DJ-1*, and* ATP13a2*) have been identified, the molecular bases of sporadic idiopathic PD remain largely unknown [3, 4]. As for other neurodegenerative diseases, such as Alzheimer's disease and Huntington's disease, ageing is considered a major risk factor for PD development [5].

The current view is that the slow and progressive death of SNpc mDA neurons remains asymptomatic until 30% of mDA neuron cell bodies and 50–60% of axonal terminals are lost [6]. Over time, this loss results in severe dopamine (DA) deficiency in the striatum, leading to the cardinal motor symptoms including rest tremor, bradykinesia, rigidity, and postural instability. No therapies are yet available to prevent the loss of mDA neurons or even delay the course of the disease [7]. One remarkable feature of the disease is that nonnigral dopaminergic neurons including mDA neurons in the ventral tegmental area (VTA), located in the vicinity of the SNpc mDA neurons, are relatively spared. The molecular determinants for the selective vulnerability of the SNpc mDA neurons in PD are not known [8, 9].

A number of studies have pointed to oxidative stress, mitochondrial dysfunction, protein misfolding and aggregation, impaired proteasomal and lysosomal degradation pathways, altered vesicular trafficking, and neuroinflammation as possible culprits in PD pathogenesis [1, 2, 8]. Many PD-linked genes affect mitochondrial activity or integrity [10] and a potential link between mitochondrial dysfunction and PD is supported by the ability of complex I-specific neurotoxins such as MPTP (1-methyl-4-phenyl-1,2,3,6-tetrahydropyridine) to induce PD-like symptoms in rodents and primates, including humans. Recent studies also suggest that DNA damage or repair dysfunction [11–13] and nucleolar stress [14–17] play important roles in PD pathogenesis and other neurodegenerative diseases [18–23]. Mouse lines expressing PD-linked gene mutations recapitulate several features of PD pathogenesis but most of them do not present the selective and progressive loss of mDA neurons in the SNpc [24–26].

Major progress has recently been made in dissecting the genetic and signalling networks that control the generation of mDA neurons [27–29]. These studies have revealed the crucial role of several transcription factors. Interestingly, a number of these transcription factors (e.g., Engrailed-1/Engrailed-2, Foxa1/2, Lmx1a/b, Nurr1, Otx2, and Pitx3) remain present in adult mDA neurons and are required for their maintenance throughout life [30–32]. The elucidation of the roles and mechanisms of action of these transcription factors during development and adulthood could bring important insights into PD pathogenesis and suggest new therapeutic strategies. It is noteworthy that possible genetic links between these transcription factors and PD have been reported [33–46]. This review summarizes some aspects of the function of these developmental transcription factors in relation to PD.

## 2. Transcription Factors as Key Players in mDA Neuron Development

Midbrain DA neuron development starts around embryonic day E8 in the mouse with the induction of neurons in the floor plate and the specification of mDA progenitors. These progenitors give rise to mature mDA neurons following successive steps of proliferation, maturation, migration, axonal pathfinding, and synaptogenesis [27–29]. Midbrain DA neurons of the SNpc project mainly to the dorsal striatum to form the nigrostriatal pathway involved in the control of voluntary movements, whereas VTA mDA neurons project to the nucleus accumbens, the amygdala, the hippocampus, and the prefrontal cortex to form the mesolimbic and mesocortical pathways involved in motivation, reward, addiction, cognition, and memory. Mature SNpc mDA neurons express elevated levels of the highly active glycosylated form of the dopamine transporter (DAT) glyco-DAT and the inwardly rectifying potassium channel GIRK2, whereas VTA mDA neurons are more enriched in the calcium binding protein calbindin D28K (CALB1) [47].

Induction and specification of DA progenitors are governed by the concerted action of secreted factors (e.g., SHH, FGF8, Wnt1, and TGF-*β*) and of key transcription factors including En1/2, Otx2, Lmx1a/b, and Foxa1/2 [27–29]. Early loss of function of any of these transcription factors has dramatic consequences in the ontogenesis of mDA neurons. The subsequent steps of mDA neuron development are accompanied by the expression of additional transcription factors such as Nurr1 and Pitx3, which participate in the differentiation of mDA progenitors into mature mDA neurons. Hence, Nurr1 and Pitx3 are required for the expression of several genes encoding proteins that determine mature mDA neuron identity such as TH (tyrosine hydroxylase), DAT and VMAT2 (vesicular monoamine transporter 2), AADC (dopa decarboxylase), DRD2 (dopamine receptor D2), or ALDH1A1 (aldehyde dehydrogenase 1 family, member A1). Furthermore, Nurr1/Pitx3 are persistently required for maintaining the adult expression of these genes and mDA neurons with a conditional adult ablation of* Nurr1* degenerate progressively [48]. Interestingly, functional interactions between pairs of transcription factors (e.g., Nurr1 and Pitx3, Pitx3 and En1/2, or Nurr1 and Foxa1/2) have been reported [49–51].

Earlier work demonstrated that En1/2 are required for the survival of mature mDA neurons during late embryonic life in a dose-dependent manner [52–54]. This might be achieved through the activation of the Erk1/2 MAPK survival pathways and suppression of the proapoptotic activity of the proneurotrophin receptor p75NTR [55]. It was also shown that En1/2 is involved in the acquisition of a mature mDA neuron identity [50]. For example, in* En1* homozygous mutants (viable on a C57BL/6 background), expression at E13.5 of* Pitx3*,* Th*,* Dat*,* Vmat2*, and* Ddc* (encoding AADC) is reduced in the rostral-lateral mDA domain [50]. Otx2, together with Sox6, also controls mDA neuron subtype identity [56] and in the course of development, its expression becomes restricted to a specific subset of dorsal-lateral VTA mDA neurons [47]. In addition to transcription regulation, the importance of epigenetic mechanisms in all these processes must also be recalled [57].

## 3. Developmental Transcription Factors Required in Adult mDA Neuron Maintenance

As mentioned above, many developmental transcription factors remain expressed in mDA neurons throughout life and are required for their survival and physiological functions. We shall now briefly describe the effects of loss or gain of function of some of these transcriptions factors and their relevance to PD in adult mDA neurons.

### 3.1. Manipulating the Expression of Nurr1, Otx2, Foxa1/2, and Pitx3 in Adult mDA Neurons

*Nurr1* expressed in adult mDA neurons of the SNpc and VTA is critical for the maintenance of their phenotype [58, 59].* Nurr1*-deficient mice die shortly after birth.* Nurr1* haplodeficient young animals present a normal number of mDA neurons and have no abnormal motor phenotype, but the number of mDA neurons decreases in old mice (after 15 months) in parallel with a decreased locomotor activity [48].* Nurr1+/−* mice also exhibit increased vulnerability to MPTP [60] and show an exacerbated sensitivity to the toxicity of repeated methamphetamine exposure [61].* Nurr1* ablation in adult mDA neurons using AAV-Cre leads to mDA neuron dysfunction and to the progressive loss of mDA neuron markers [48]. Finally, tamoxifen-induced conditional deletion of* Nurr1* in mDA neurons in 5-week-old mice results in a progressive pathology, associated with loss of or reduced striatal DA, impaired motor behaviour, and dystrophic axons and fragmented dendrites containing varicosities [62]. However, no major loss of mDA neurons was reported in these mice.

*Otx2* is expressed in a subset of mDA neurons in the central and mediolateral area of the VTA in the adult [47]. Conditional knockout of* Otx2* in the adult leads to selective loss of the axonal projections from VTA mDA neurons [63, 64]. Otx2 is also a negative regulator of DAT and there is an inverse correlation between* Otx2* expression and glyco-DAT levels in mDA neurons [47]. Otx2 gain of function in SNpc mDA neurons decreases glyco-DAT levels, thus conferring protection against MPTP toxicity [47, 65].* Foxa1/2* also continue to be expressed in adult mDA neurons and* Foxa2* heterozygous mice present late-onset, spontaneous degeneration of mDA neurons [66]. Conditional tamoxifen-inducible deletion of both* Foxa1* and* Foxa2* in early adulthood results in a decline of striatal DA content along with locomotor deficits and progressive loss of ALDH1A1, AADC, and DAT, ultimately leading to a reduction of mDA neurons in the SNpc of aged animals [67]. Finally, the spontaneous deletion of* Pitx3* in the Aphakia mouse or global Pitx3 gene inactivation leads to rapid and preferential loss of mDA neurons in the SNpc of neonatal mice [68, 69]. Dorsal SNpc mDA neurons, which do not express Pitx3, are spared in mutant mice similar to what is observed in PD [70].

### 3.2. En1 Heterozygous Mice as a Model for PD

*En1/2* are expressed in SNpc and VTA mDA neurons from early development on into adulthood [52]. Although* En1−/−* pups die (OF1 background) at birth [71],* En1* heterozygous mice are viable.* En1+/−* mice display a normal number of mDA neurons until 6 weeks after birth when SNpc mDA neurons start to die progressively [72]. The extent of cell death reaches about 40% in the SNpc at 48 weeks of age and is correlated with a decreased DA content in the striatum. Midbrain DA neurons in the VTA are affected to a much lesser extent.* En1+/−* mice present PD-like motor symptoms such as decreased spontaneous locomotor activity (distance travelled, rearing), increased amphetamine-sensitization, and decreased motor coordination and sensorimotor learning (rotarod). The loss of mDA neurons in the VTA, albeit less pronounced, also leads to some nonmotor behaviour alterations such as increased depressive-like behaviour (forced swimming test), increased anhedonic-like behaviour (saccharine preference), and poor social interaction [72]. This suggests that the mesolimbic system is also affected in these mutants. The death of adult mDA neurons from* En1* haploinsufficiency has now been observed in several independent studies [50, 73, 74] and follows the retrograde degeneration of axons [50, 73, 74].* En1+/−*;* En2−/−* mice (C57BL/6 background) are normal at birth but present a massive loss of mDA neurons in the SNpc of young adult, illustrating an En1/2 dosage effect on survival [75]. Midbrain DA neurons in these mice are also more responsive to MPTP-induced cell death.

A more detailed characterization of* En1* heterozygous mice revealed early signs of degeneration of mDA axon terminals in the striatum [74], prior to neuronal cell loss in the SNpc. Dopaminergic terminals become dystrophic and swollen, contain autophagic vacuoles, and present deficits in DA release and uptake. The nigral dopaminergic cell bodies exhibit signs of decreased autophagy accompanied by an increase in mTOR activity and a decrease of the autophagic marker LC3B [74]. These findings illustrate a retrograde degeneration of the nigrostriatal system in* En1+/− *mice, akin to what occurs in PD [6, 76, 77]. Retrograde degeneration may be a common feature of many progressive neurodegenerative disorders [78]. Individual axons in the nigrostriatal pathway of* En1+/−* mice undergo fragmentation supporting the idea that axonal transport failure might be an early feature of PD [79]. The possible role of autophagy in PD pathogenesis [80, 81] was recently assessed in a mouse model generated by the conditional deletion of the autophagy-related gene* Atg7*, which recapitulates many pathologic features of PD, including age-related loss of mDA neurons [82].* En1* heterozygous mice thus represent a valuable model to gain further insights into PD pathogenesis.

A recent study shows that mDA neurons in* Lmx1b* conditional knockout mice are progressively lost, in both the SNpc and the VTA. These mice also present abnormally large nerve terminals in the striatum and these terminals are filled with autophagic and lysosomal vesicles, before the onset of mDA cell loss. Very much in analogy with the* En1+/−* mouse phenotype, these findings suggest a retrograde degeneration of mDA neurons. Alteration of the autophagy/lysosomal pathway could be due to increased mTOR activity of mDA neurons in* Lmx1b* mutants and in* En1* heterozygous mice. In this context it is of note that rapamycin treatment of conditional* Lmx1b* knockout mice normalizes the phenotypic alterations [83]. Finally, gene expression profiling in the MN9D dopaminergic cell line identified nuclear-encoded mitochondrial subunits of the respiratory chain as potential* Lmx1a* targets, suggesting a possible link also between* Lmx1a* and mitochondria [84].

## 4. Developmental Transcription Factors and Neuroprotective Approaches for PD

### 4.1. Protection of mDA Neurons by En1/2 Protein Transduction in Experiential PD Models

It is now well established that several homeoproteins, including En1/2 and Otx2, are endowed with the ability to transduce cells [85, 86]. This property was exploited to examine the therapeutic potential of En1/2. It was first shown that mDA neuronal loss in* En1+/−* mice can be stopped by infusing recombinant En1/2 proteins (En1 and En2 are biochemically equivalent) in the SNpc [72]. Subsequently, En1/2 protein transduction was shown to protect mDA neurons in various experimental models of PD* in vitro* and* in vivo*, including the MPTP, rotenone, 6-OHDA (6-hydroxydopamine), and mutated a-synuclein (A30P) models [87]. Interestingly, unilateral Engrailed infusion in naive mice increases ipsilateral striatal DA content. This results in amphetamine-induced turning behaviour contralateral to the side of infusion indicating an activation of the nigrostriatal pathway upon Engrailed infusion in the SNpc [87]. Thus, En1/2 is able not only to protect mDA neurons against various PD-related insults but also to increase their physiological activity. Finally, it was shown that forced expression of* Otx2* in mDA neurons in the SNpc of* En1+/−* mice can prevent the progressive loss of mDA neurons caused by* En1* haploinsufficiency [73]. Ectopic expression of* Otx2* in SNpc mDA neurons also protects them against MPTP toxicity (see above). Otx2 protein transduction could thus also be of potential therapeutic interest for neuroprotection in PD. It is noteworthy that Otx2 protein transduction has previously been shown to protect retinal ganglion cells (RGCs) against NMDA (N-methyl-D-aspartate) toxicity in a mouse model of glaucoma [88].

### 4.2. Use of Developmental Transcription Factors for Cell Replacement Strategies

Although the feasibility of cell replacement therapy for PD has been demonstrated with embryonic ventral midbrain tissue transplantation [89], the scarcity of the material for transplantation remains a major hurdle [90]. The knowledge gained from the genes and mechanisms involved in mDA neuron development has been very valuable for the generation of midbrain DA progenitors from embryonic stem cells (ESCs) or induced pluripotent stem cells (iPSCs) [91]. ESCs and iPSCs converted into DA progenitors expressing* Lmx1a* and* Foxa2* by exposure to SHH and FGF8 (with or without Wnt1/TGF-*β*1/retinoic acid) can then differentiate into DA neurons* in vitro* and/or* in vivo* [92–94]. Similarly,* Nurr1* expression together with that of the transcription factor Ascl1 (also important for mDA progenitor specification) is sufficient to drive DA differentiation of forebrain embryonic rat neural precursors [95–97]. Proper innervation of target areas and functional recovery upon transplantation of* in vitro* generated DA progenitors has now been demonstrated in both rodents and nonhuman primate PD models [98–101]. To further analyse the functionality of transplanted cells, two recent studies used optogenetic tools or “designer receptor exclusively activated by designer drug” (DREADD) technology to stimulate the function of engrafted DA neurons* in vivo* by illumination or by injecting specific drugs, respectively [102–104]. Such approaches will be particularly useful to assess the long-term function of transplanted cells in PD models.

From a safety point of view, as an alternative to DNA or RNA mediated gene delivery, a few studies have considered protein transduction as a means for reprogramming through the delivery of cocktails of recombinant proteins fused to cell penetrating peptides (CPPs) [85, 105]. It was reported that protein-based human iPSCs can efficiently generate functional dopamine neurons and can treat a rat model of Parkinson disease [106]. The neuroprotection achieved by transduction of En1/2 or Otx2 [72, 87, 88], two homeoproteins naturally containing the “penetratin” sequence, should encourage more direct protein delivery-based strategies for neuroprotection or neurorepair.

## 5. Mechanisms of Action of Developmental Transcription Factors in Adult mDA Neurons

**Figure 1:**
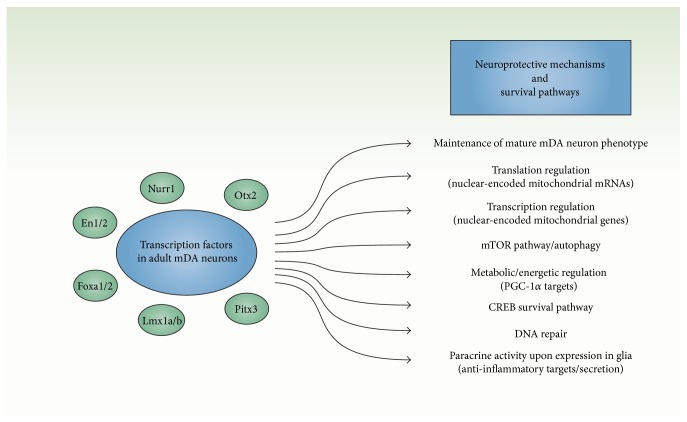
Multiple mechanisms and signalling pathways engaged by transcription factors for neuroprotection of adult mDA neurons.

Our knowledge concerning the mechanisms of action of these developmental transcription factors in the survival and maintenance of adult mDA neurons is still limited. However, it has emerged from recent studies that these transcription factors engage several neuroprotective mechanisms and are linked to several survival pathways in adult mDA neurons (Figure 1). Since Otx2 gain of function in the SNpc can prevent cell loss in* En1+/−* mice [73], it is likely that Otx2 and En1/2 share some common neuroprotective mechanisms.

Engrailed survival activity relies on several mechanisms and signalling pathways. It is noteworthy that in addition to being a transcription factor Engrailed is also a translation regulator [31] that guides retinal axons through the translation of local mRNAs [107–110]. This property is explained by the fact that Engrailed, like many other homeoproteins, interacts with the translation initiation factor eIF4E and regulates cap-dependent translation [111, 112]. In the context of neuron survival, it was shown that Engrailed protects mDA neurons against MPTP by upregulating the translation of a subset of nuclear-encoded mitochondrial complex I subunits (e.g., Ndufs1/Ndufs3), thus enhancing complex I activity and ATP synthesis [87, 109]. The importance of translational regulation of nuclear-encoded mitochondrial mRNAs coding for respiratory chain components has also been underscored recently for the function of some PD-linked genes [113]. All these studies support the idea that a failure to sustain the high-energy demand of mDA neurons may be critical in PD pathogenesis [114, 115].

Mitochondria are critically required for long-term axonal survival and maintenance [116]. Thus, decreased mitochondrial activity might contribute to retrograde cell death in* En1* heterozygous mice. As mentioned above, Engrailed plays a role in the activation of the mTOR pathway and the regulation of autophagy [74, 107]. The search for En1/2 translation targets in retinal axons of the Xenopus also identified Lamin B2, which is a major constituent of the nuclear envelope. It was shown that Lamin B2 translation in axons regulates mitochondrial size and mitochondrial membrane potential and supports axon survival [110]. En1/2 might thus play a role in mitochondrial activity and axon maintenance throughout adulthood.

Nurr1-mediated survival might involve neurotrophic GDNF/Ret signalling since Ret is a Nurr1 target gene [117, 118]. Nurr1 is downregulated by mutated *α*-synuclein [119, 120] and this could compromise GDNF/Ret survival signalling in PD. Absence of Ret signalling in mice causes progressive and late degeneration of the nigrostriatal system [121]. A recent study shows that Parkin cooperates with GDNF/Ret signalling to improve mitochondrial function through activation of the prosurvival NF-*κ*B pathway and prevents mDA neuron degeneration [122]. Gene expression profiling in adult conditional* Nurr1* knockout mice also identified several nuclear-encoded mitochondrial genes as potential Nurr1 transcriptional targets [62]. Thus the mitochondria appear to be a target of Nurr1 activity for mDA neuron maintenance in the adult. In addition, Nurr1 was reported to be a downstream target of the cAMP response element binding protein (CREB) mediated neuroprotection [123]. Another function of Nurr1 in the nucleus might be related to DNA double strand break repair [124]. Interestingly, Nurr1 expression is induced in microglia and astrocytes under inflammatory conditions. Nurr1 activity in these cells suppresses proinflammatory NF-*κ*B target gene expression through recruitment of the CoREST corepressor complex [125]. A more recent study shows that forced expression of Nurr1 and Foxa2 in glial cells markedly protects mDA neurons in the MPTP mouse model of PD [126]. Nurr1 and Foxa2 act synergistically in microglia to decrease the production and release of proinflammatory cytokines and enhance the synthesis and secretion of neurotrophic factors (e.g., GDNF, BDNF, NT3, SHH, erythropoietin, thioredoxin, TGF-*β*, and IGF-1) with paracrine action on mDA neurons [126]. In view of the ability of homeoproteins to be secreted and internalized, it will be interesting to examine if En1/2 and Otx2 play similar roles in glial cells in a non-cell-autonomous manner. The role of non-cell-autonomous signalling by these homeoproteins has been extensively demonstrated in axon guidance in the visual system for En1/2 [107–109] and in visual cortex plasticity for Otx2 [127, 128].

Pitx3 targets are also linked to several survival pathways in mDA neurons. The Pitx3 target* Aldh1a1* is crucial for the production of retinoic acid that exerts antiapoptotic and antioxidant activities.* Aldh1a1* is expressed in a subpopulation of mDA neurons in the SNpc and VTA. As already mentioned, the dependence on Pitx3 is not uniform for all mDA neurons. In* Pitx3* hypomorphic* Aphakia* mutants, a subpopulation of Pitx3-deficient neurons persists and these neurons are less vulnerable to MPTP-induced degeneration [129]. It has been suggested that striatal uptake and retrograde axonal transport of GDNF maintains proper expression of* Pitx3* and its target* Bdnf* in SNpc mDA neurons [130]. BDNF functions in synaptic transmission, plasticity, and growth and might contribute to synaptic maintenance of the nigrostriatal mDA neurons throughout adulthood [131]. A potential link between Pitx3 and PGC-1*α* (peroxisome proliferator-activated receptor gamma, coactivator 1 *α*), which is a positive regulator of genes required for mitochondrial biogenesis and cellular antioxidant responses, has also been recognized [132]. Overexpression of* PGC-1α* disrupts mitochondrial activity end energy balance and this might partly be due to downregulation of* Pitx3* by* PGC-1α* [133]. Finally, Pitx3 also regulates microRNA miR-133b expression, which in turn downregulates* Pitx3* [134].* miR-133b* was shown to be downregulated in PD patients but the exact role of miR-133b in mDA neuron survival is not known [134].

## 6. Perspectives: From Basic Science to Potential New Therapeutic Avenues for PD

The characterization of mDA neuron populations in the SNpc and VTA, based on the expression of selected markers [9] and more recent single cell gene expression profiling [135], has revealed a substantial heterogeneity across neurons. This suggests that selected sets of transcription factors expressed in mDA neuron subpopulations might determine the degrees of vulnerability in PD. These developmental transcription factors also have adult functions through the regulation of mitochondrial activity and several survival signalling pathways. Disruption of postmitotic neuron maintenance through an alteration of their transcriptional/translational regulation may lead to neurodegeneration [136, 137], often marked by cell cycle entry prior to death [138, 139]. As suggested above, many physiological processes participating in neuronal health and survival are controlled by developmental transcription factors. Possible sites of action are DNA repair and chromatin remodelling as well as pathways controlling genome stability. A recent example is Tau-mediated promotion of neurodegeneration through global heterochromatin relaxation [140]. Indeed these are homeostatic processes that can be regulated by classical signalling pathways as shown in the case of SHH [141]. From a more practical point of view, the successful use of homeoproteins, which have the innate ability to transduce, in the protection of mDA neurons has emphasized the potential of protein transduction-based strategies to deliver proteins directly into the cells of interest [142, 143]. The development of therapeutic proteins endowed with their own transduction domain, as is the case for homeoproteins, or made cell-permeable by the addition of a CPP-tag, could thus be seen as an alternative to cell grafting or gene therapy, provided that their effects be long-lasting.

## Abbreviations

AADC: Dopa decarboxylase (aromatic L-amino acid decarboxylase)
AAV: Adenoassociated virus
ALDH1A1: Aldehyde dehydrogenase 1 family, member A1
Ascl1: Achaete-scute family bHLH transcription factor 1
Atg7: Autophagy-related 7
ATP13a2: ATPase type 13A2
BDNF: Brain-derived neurotrophic factor
CALB1: Calbindin 1, 28 kDa
CPP: Cell penetrating peptide
CREB: cAMP-responsive-element-binding protein
DA: Dopamine
DAT: Dopamine transporter (SLC6A3)
DDC: Gene encoding AADC
DJ-1: Parkinson protein 7 (PARK7)
DRD2: Dopamine receptor D2
DREADD: Designer receptors exclusively activated by designer drugs
eiF4E: Eukaryotic translation initiation factor 4E
En1/2: Engrailed-1/Engrailed-/2
Erk1/2: Extracellular signal-regulated-kinase 1/2
ESC: Embryonic stem cell
FGF8: Fibroblast growth factor 8
Foxa1/2: Forkhead box A1/2
GDNF: Glial cell derived neurotrophic factor
GIRK2: Potassium inwardly rectifying channel, subfamily J, member 6 (KCNJ6)
IGF-1: Insulin-like growth factor 1
iPSC: Induced pluripotent stem cells
LC3B: Microtubule-associated protein 1 light chain 3 beta (MAP1LC3B)
Lmx1a/b: LIM homeobox transcription factor 1, alpha/beta
LRRK2: Leucine-rich repeat kinase 2
MAPK: Mitogen-activated protein kinase
mDA: Midbrain dopaminergic
MPTP: 1-Methyl-4-phenyl-1,2,3,6-tetrahydropyridine
mTOR: Mechanistic target of rapamycin (serine/threonine kinase)
Ndufs1/3: NADH dehydrogenase (ubiquinone) Fe-S protein 1/3
NF-*κ*B: Nuclear factor of kappa light polypeptide gene enhancer in B-cells
NMDA: N-Methyl-D-aspartate
Nurr1: Nuclear receptor subfamily 4, group A, member 2 (Nr4a2)
NT3: Neurotrophin 3 (NTF3)
6-OHDA: 6-Hydroxydopamine
Otx2: Orthodenticle homeobox 2
p75NTR: Nerve growth factor receptor (NGFR)
PARKIN: Parkin RBR E3 ubiquitin protein ligase (PARK2)
PD: Parkinson disease
PGC-1*α*: Peroxisome proliferator-activated receptor gamma, coactivator 1 *α*
PINK1: PTEN induced putative kinase 1
Pitx3: Paired-like homeodomain 3
Ret: Ret protooncogene
RGC: Retinal ganglion cells
SHH: Sonic hedgehog
SNCA: Synuclein, *α*
SNpc: Substantia nigra pars compacta
Sox6: SRY- (sex determining region Y-) box 6
Tfam: Transcription factor A, mitochondrial
TGF-*β*: Transforming growth factor, *β* 1
TH: Tyrosine hydroxylase
VMAT2: Vesicular monoamine transporter 2 (SLC18A2)
VTA: Ventral tegmental area
Wnt1: Wingless-type MMTV integration site family, member 1.
